# Attenuation Effect of Salvianolic Acid B on Testicular Ischemia-Reperfusion Injury in Rats

**DOI:** 10.1155/2022/7680182

**Published:** 2022-01-13

**Authors:** Si-Ming Wei, Yu-Min Huang

**Affiliations:** ^1^Shulan International Medical College, Zhejiang Shuren University, Hangzhou City, Zhejiang Province 310015, China; ^2^School of Nursing, Zhejiang Chinese Medical University, Hangzhou City, Zhejiang Province 310053, China; ^3^Department of Sport Science, College of Education, Zhejiang University, Hangzhou City, Zhejiang Province 310058, China

## Abstract

During testicular ischemia-reperfusion, overproduction of reactive oxygen species is associated with testicular injury. We injected hydrogen peroxide (a representative of reactive oxygen species) into normal testis via the testicular artery. The experiment demonstrates that reactive oxygen species can cause spermatogenic injury. Salvianolic acid B, the most abundant bioactive component in *Salvia miltiorrhiza* Bunge, has been reported to possess a potent antioxidant activity. This study was conducted to evaluate the effect of salvianolic acid B on testicular ischemia-reperfusion injury in a rat testicular torsion-detorsion model. Rats were randomly separated into three groups, including 20 rats in each group: control group with sham operation, testicular ischemia-reperfusion group, and testicular ischemia-reperfusion + salvianolic acid B-treated group. In the testicular ischemia-reperfusion group, left testicular torsion of 720° for 2 hours was induced, and then testicular detorsion was carried out. Rats in the salvianolic acid B-treated group additionally had salvianolic acid B administered intravenously at detorsion. At 4 hours after detorsion, testes of 10 rats from each group were collected to analyze the protein expression of xanthine oxidase which catalyzes generation of reactive oxygen species and malondialdehyde concentration (an indirect indicator of reactive oxygen species). At 3 months after detorsion, testes of the remaining 10 rats from each group were collected to analyze spermatogenesis. Compared with the control group, xanthine oxidase protein expression and malondialdehyde concentration in ipsilateral testes of testicular ischemia-reperfusion group increased significantly, while spermatogenesis decreased significantly. In the salvianolic acid B-treated group, xanthine oxidase protein expression and malondialdehyde concentration in ipsilateral testes decreased significantly, while spermatogenesis increased significantly, compared with the testicular ischemia-reperfusion group. These results suggest that salvianolic acid B can attenuate testicular torsion/detorsion-induced ischemia/reperfusion injury by downregulating the xanthine oxidase protein expression to inhibit reactive oxygen species formation.

## 1. Introduction

Testicular torsion was first described by Delasiavue in 1840 [1]. It is caused by the twisting of the spermatic cord, which results in decreased blood flow to the testis. Therefore, testis is in an ischemic condition after testicular torsion. If this condition goes on for more than six hours, testicular infarction will occur [2, 3]. Surgical detorsion is critical for providing blood supply to the testis. Even if testicular detorsion is performed timely, testicular atrophy on follow-up examination has been reported in clinical studies [4–6]. The mechanism underlying testicular damage during torsion-detorsion is ischemia-reperfusion injury. Although the exact mechanisms of the injury have not been completely understood, excess reactive oxygen species produced during the ischemia-reperfusion play an important role in tissue damage [7–10]. Reactive oxygen species (mainly hydrogen peroxide, superoxide anion, and hydroxyl radical) cause tissular damage by oxidizing cellular membrane lipids, deoxyribonucleic acid (DNA), and proteins [11].

To date, treatment for testicular ischemia-reperfusion injury is completely absent in the clinical setting. *Salvia miltiorrhiza* Bunge, also called Danshen in China, is a well-known traditional Chinese herb and officially listed in the Chinese Pharmacopoeia [12]. It has been widely used for thousands of years to treat cardiovascular and cerebrovascular diseases in China and other Asian countries [13, 14]. Salvianolic acid B is the most abundant bioactive component in *Salvia miltiorrhiza* Bunge and has been regarded as a marker ingredient of *Salvia miltiorrhiza* Bunge in the Chinese Pharmacopoeia [15]. Its molecular formula and molecular weight are C_36_H_30_O_16_ and 718.62 Dalton, respectively [13]. In structure, salvianolic acid B is composed of 3 molecules of tanshinol and 1 molecule of caffeic acid [16]. Salvianolic acid B has been reported to possess various pharmacological activities, such as antioxidation, anti-inflammation, antifibrosis, and anticancer [17, 18]. Many studies have demonstrated that salvianolic acid B can protect against ischemia-reperfusion injury in different organs, including heart, brain, liver, kidney, spinal cord, and skin flap [19–24]. To our knowledge, the effect of salvianolic acid B on testicular ischemia-reperfusion injury has not been investigated yet. Thus, the current study is aimed at evaluating the efficacy of salvianolic acid B in attenuating testicular ischemia-reperfusion injury in the rat testicular torsion-detorsion model.

## 2. Materials and Methods

### 2.1. Animals

One hundred male Sprague-Dawley rats (250-300 g, 8 weeks old) were obtained from Shanghai SLAC Laboratory Animal Co., Ltd. (Shanghai City, China). Sixty of the 100 rats were used for testicular ischemia-reperfusion experiment. The other forty rats were used for hydrogen peroxide-injected experiment. Animals were kept in standard conditions of a temperature (21°C ± 1°C), relative humidity (55% ± 5%), and 12 h light/12 h dark cycle. Rats had free access to standard pellet diet and water during the experimental period. All animal experiments were carried out with the approval of Local Animal Ethics Committee (Approval No. 10790).

### 2.2. Surgical Procedure

The rats were randomly divided into three groups of twenty rats per group, group 1: sham-operated control; group 2: testicular ischemia-reperfusion; and group 3: testicular ischemia-reperfusion + salvianolic acid B treatment. The surgical procedure was performed according to our previously described method [25]. An intraperitoneal injection of ketamine (50 mg/kg; Sigma Chemical Company, St. Louis, MO, USA) was used for general anesthesia in each rat. The animals breathed spontaneously throughout all surgical procedures. The surgical area was shaved and scrubbed with povidone-iodine solution. The left testis was exteriorized through a left-sided ilioinguinal incision. A 11-0 atraumatic silk suture was placed through tunica albuginea in the control group. The testis was immediately placed back into its scrotal position, and a 4-0 silk suture was used to close the incision. The left testes of ischemia-reperfusion group rats were twisted 720° counterclockwise to establish testicular ischemia. The testes were attached to the scrotum with 11-0 silk suture in order to keep testicular ischemic state. The ischemia period was two hours, and then the reperfusion was performed by counterrotating the torsional left testis to its normal position. The testis was still living and was returned to the scrotum. In the salvianolic acid B-treated group, after performing the same surgical procedure as in the ischemia-reperfusion group, salvianolic acid B (10 mg/kg; Sigma Chemical Company) was given via the tail vein at the starting time of reperfusion. The dose of salvianolic acid B was chosen in our study based on previous studies [19–21, 23]. We removed the left and right testes of ten rats from each group four hours after reperfusion for determination of xanthine oxidase protein expression and malondialdehyde level. Then, these rats were sacrificed by the carbon dioxide method. Bilateral testes of the remaining 10 rats from each group were excised three months after reperfusion for evaluation of testicular spermatogenesis.

### 2.3. Immunoblotting Assay for the Xanthine Oxidase Protein Expression

Testicular tissue (100 mg) was homogenized with a glass homogenizer in 1 ml ice-cold lysis buffer (ingredients: 50 mM Tris HCl, pH 7.4, 1 mM phenylmethylsulfonyl fluoride, 1% nonidet P-40, 2 mM sodium orthovanadate, 0.5 *μ*g/ml leupeptin, 150 mM NaCl, 5 *μ*g/ml aprotinin, 0.1% sodium dodecyl sulfate, 0.5% sodium deoxycholate, 0.5 mM ethylenediaminetetraacetic acid, and 1 mM dithiothreitol) for 15 minutes. The homogenate was incubated for 30 minutes on ice and then centrifuged at 14,000 × gravity for 15 minutes at 4°C. The supernatant was collected and used to quantify protein concentration by the Bradford method [26]. The protein sample was denatured at 100°C for 3 minutes in loading buffer. The loading buffer contained 0.004% bromphenol blue, 10% 2-mercaptoethanol, 20% glycerol, 4% sodium dodecyl sulfate, and 0.125 M Tris HCl, pH approximate 6.8. Same amount of protein (20 *μ*g) from each sample was loaded into each well and fractionated by sodium dodecyl sulfate-polyacrylamide gel electrophoresis. The gel-separated proteins were electrophoretically transferred onto a nitrocellulose membrane which was subsequently blocked for 1 hour at room temperature in a blocking solution containing 5% skim-milk powder to prevent non-specific antibody binding. Then, the membrane was subjected to incubation with primary antibodies against xanthine oxidase (Santa Cruz Biotechnology, Santa Cruz, CA, USA) or *β*-actin (Sigma Chemical Company) at 4°C overnight. The *β*-actin served as an internal control. The membrane was washed three times in Tris-buffered saline containing 0.1% Tween-20 and further incubated for 1 hour at room temperature with secondary antibody tagged with horseradish peroxidase (Santa Cruz Biotechnology). After washing with Tris-buffered saline containing 0.1% Tween-20, target proteins on the membrane were developed by using a commercially available enhanced chemiluminescence reagent (Santa Cruz Biotechnology). The intensity of protein bands was quantified using a GS-700 imaging densitometer (Bio-Rad Laboratories, Hercules, CA, USA). The intensity ratio of xanthine oxidase band to internal control *β*-actin band from the same sample showed a relative protein expression level of xanthine oxidase.

### 2.4. Evaluation of Thiobarbituric Acid-Reactive Species in Testis

Testicular sample was collected and placed into an ice-cold malondialdehyde lysis buffer. Then, the sample was homogenized by a glass homogenizer followed by centrifugation at 5,000 × gravity for 15 minutes at 4°C. The supernatant was retained for malondialdehyde measurement. The protein content in the supernatant was determined by the Bradford method [26]. Malondialdehyde level in the testis was detected by the thiobarbituric acid reactive substance assay using a commercial kit (Nanjing Jiancheng Bioengineering Institute, Nanjing City, China) according to the manufacturer's protocol [27]. Tissue malondialdehyde level was calculated as nmol/mg protein.

### 2.5. Analysis in Testicular Spermatogenesis

Testicular spermatogenic evaluation was conducted as described previously [25]. Testicular weight, seminiferous tubular diameter, germ cell layer number, and Johnsen's score were used to evaluate testicular spermatogenesis. The excised testis was weighed and fixed in Bouin's solution. The fixed tissue was dehydrated through 80%, 95%, and 100% alcohol series. Then, it was made transparent through the xylene solution. Tissue specimen was processed for paraffin embedding and cut into 5 *μ*m thick paraffin section. After section was deparaffinized with xylene, and hydrated in a decreasing concentration alcohol series, it was stained by hematoxylin and eosin (Sigma Chemical Company). Testicular section was observed under a light microscope by an examiner who was blinded to the study. A total of twenty seminiferous tubules from each testis that were round or nearly round were randomly chosen to measure seminiferous tubular diameter, germ cell layer number, and Johnsen's score. Seminiferous tubular diameter was evaluated in testicular section with the help of an eyepiece micrometer. We measured the number of germ cell layers in each tubule by counting the germ cell layer numbers from basal membrane to tubular lumen at 90°, 180°, 270°, and 360°, and mean value was calculated. Histological changes in seminiferous tubule were quantified using Johnsen's scoring criteria [28]. Each seminiferous tubule was given a score on a scale of 1—10 in view of maturation rate of germinal epithelial cells. A score of 1 means neither germ cells nor Sertoli cells in the seminiferous tubule. A score of 10 means complete spermatogenesis with many spermatozoa, organized germinal epithelium with a regular thickness, and an open tubular lumen.

### 2.6. Testicular Injection of Hydrogen Peroxide

Forty rats were randomly separated into two experimental groups (*n* = 20 per group): normal saline-injected and hydrogen peroxide-injected groups. Anesthesia, disinfection, and left testicular exposure were performed as described above. In the normal saline-injected group, normal saline in a 1 ml volume was injected into left testis via the testicular artery. In the hydrogen peroxide-injected group, hydrogen peroxide (140 mM; Sigma Chemical Company) in a 1 ml volume was injected into left testis via the testicular artery. Then, left testis was placed back into the scrotum, and the incision was closed. We removed left testes of ten rats from each group four hours after injection for determination of malondialdehyde level, and these rats were finally euthanized by the carbon dioxide method. The left testes of the remaining ten rats from each group were excised three months after injection for evaluation of testicular spermatogenesis. Testicular malondialdehyde level and spermatogenesis were analyzed as described above.

### 2.7. Statistical Analysis

All results were presented as arithmetic mean ± standard deviation. Analysis of data was carried out using GraphPad Prism 4.0 statistical software (GraphPad Software Inc., San Diego, CA, USA). One-way analysis of variance and a post hoc Student-Newman-Keuls test were applied to assess the differences among all three groups. Data between ipsilateral and contralateral testes within same group were compared using Students *t*-test. Differences between normal saline-injected and hydrogen peroxide-injected groups were analyzed by Students *t*-test. The difference was considered statistically significant if *P* value was less than 0.05.

## 3. Results

### 3.1. Effect of Salvianolic Acid B on the Testicular Xanthine Oxidase Protein Expression in Rats Exposed to Testicular Ischemia-Reperfusion

**Figure 1:**
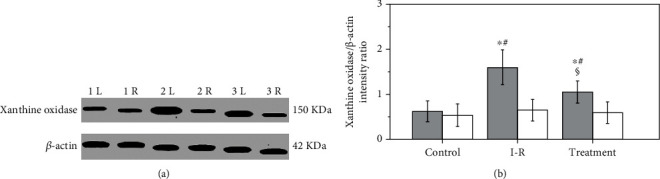
Testicular xanthine oxidase protein expression in control, ischemia-reperfusion (I-R), and salvianolic acid B-treated groups. (a) Xanthine oxidase protein expression is measured by Western blot. The *β*-actin is deemed as an internal control. Lanes 1L and 1R indicate left (i.e., ipsilateral) and right (i.e., contralateral) testes in the control group. Lanes 2L and 2R indicate ipsilateral and contralateral testes in the I-R group. Lanes 3L and 3R indicate ipsilateral and contralateral testes in the salvianolic acid B-treated group. (b) Quantitative analysis of the xanthine oxidase protein expression. The intensity ratio of xanthine oxidase band to internal control *β*-actin band from the same sample shows a relative protein expression level of xanthine oxidase. Grey bars represent ipsilateral testes; open bars represent contralateral testes. All quantitative data are displayed as mean ± standard deviation (*n* = 10 each group). ^∗^*P* < 0.01, compared with the control group; #*P* < 0.001, compared with contralateral testes in the same group; §*P* < 0.001, compared with ipsilateral testes in the I-R group.

Testicular xanthine oxidase protein expression in control, ischemia-reperfusion, and salvianolic acid B-treated groups is presented in Figure 1. Testicular ischemia-reperfusion induced a significant increase in xanthine oxidase protein expression in ipsilateral testes versus the control group (*P* < 0.001). Salvianolic acid B treatment significantly decreased the xanthine oxidase protein expression in ipsilateral testes compared with those in ischemia-reperfusion group (*P* < 0.001). No significant difference was observed in the xanthine oxidase protein expression in contralateral testes from all three groups (*P* = 0.5979).

### 3.2. Effect of Salvianolic Acid B on Testicular Malondialdehyde Level in Rats Exposed to Testicular Ischemia-Reperfusion

**Figure 2:**
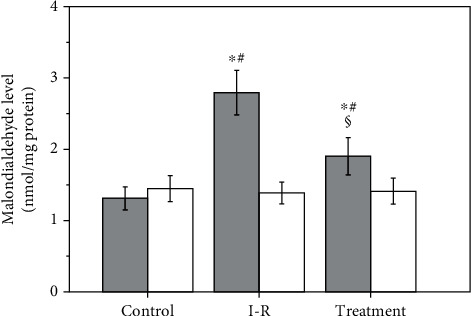
Testicular malondialdehyde level in control, ischemia-reperfusion (I-R), and salvianolic acid B-treated groups. Grey bars represent ipsilateral testes; open bars represent contralateral testes. All quantitative data are displayed as mean ± standard deviation (*n* = 10 each group). ^∗^*P* < 0.001, compared with the control group; #*P* < 0.001, compared with contralateral testes in the same group; §*P* < 0.001, compared with ipsilateral testes in the I-R group.

As shown in Figure 2, malondialdehyde level in the ischemia-reperfusion group was significantly higher in ipsilateral testes than that in control group (*P* < 0.001). The malondialdehyde level in the salvianolic acid B-treated group was significantly lower in ipsilateral testes than that in the ischemia-reperfusion group (*P* < 0.001). All three groups displayed a nonsignificant difference in malondialdehyde level in contralateral testes (*P* = 0.7338).

### 3.3. Effect of Salvianolic Acid B on Testicular Spermatogenesis in Rats Exposed to Testicular Ischemia-Reperfusion

**Figure 3:**
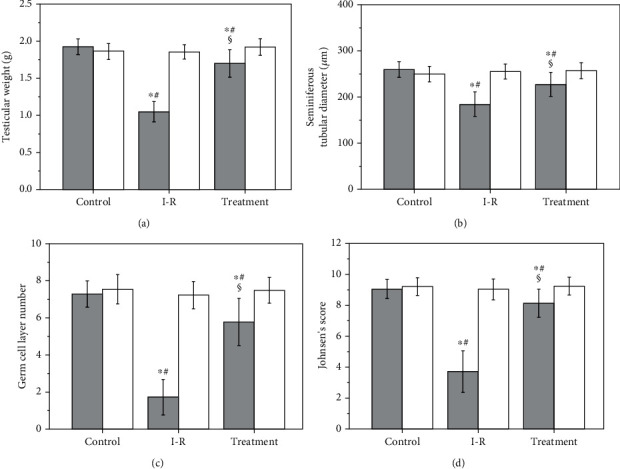
Testicular weight (a), seminiferous tubular diameter (b), germ cell layer number (c), and Johnsen's score (d) in control, ischemia-reperfusion (I-R), and salvianolic acid B-treated groups. Grey bars represent ipsilateral testes; open bars represent contralateral testes. All quantitative data are displayed as mean ± standard deviation (*n* = 10 each group). ^∗^*P* < 0.05, compared with the control group; #*P* < 0.01, compared with contralateral testes in the same group; §*P* < 0.001, compared with ipsilateral testes in the I-R group.

**Figure 4:**
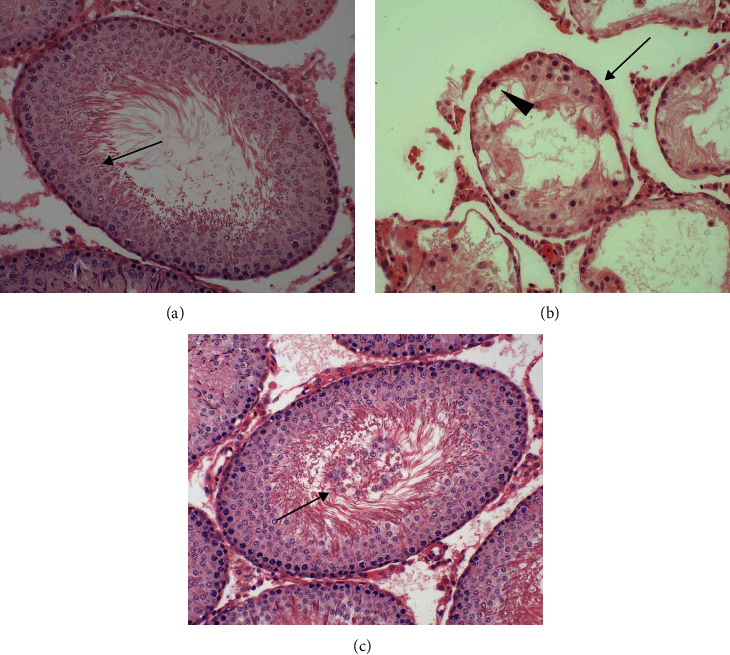
Light microscopy of hematoxylin and eosin stained sections of rat testicular tissues in control, ischemia-reperfusion, and salvianolic acid B-treated groups (original magnification, ×200). (a) Bilateral testes in the control group and contralateral nontorsional testes in ischemia-reperfusion and salvianolic acid B-treated groups all exhibited normal diameter of seminiferous tubule, germ cell layer number, and spermatogenesis from spermatogonia in tubular basal membrane to mature spermatozoa (↑) in tubular lumen. The germinal epithelium of seminiferous tubule left an open lumen in tubular center. (b) The diameter of seminiferous tubule (↑) and germ cell layer number (▲) were lower in ipsilateral torsional testes of ischemia-reperfusion group versus control group (*P* < 0.001). Mature spermatozoa were completely absent in seminiferous tubule. (c) Ipsilateral testes in the salvianolic acid B-treated group showed that histologic structure of seminiferous tubule was close to normal. However, in tubular lumen, there were many sloughed germinal cells (↑), which easily blocked up tubular lumen.

As shown in Figures 3 and 4, testicular weight, seminiferous tubular diameter, germ cell layer number, and Johnsen's score in ipsilateral testes were found to be significantly lower in the ischemia-reperfusion group compared with the control group (*P* < 0.001). A significant increase occurred in the four parameters of ipsilateral testes in the salvianolic acid B-treated group compared with those in the ischemia-reperfusion group (*P* < 0.001). The difference among three groups in term of the four parameters was not significant in contralateral testes (*P* = 0.3476, *P* = 0.6066, *P* = 0.6054, and *P* = 0.7252, respectively).

### 3.4. Effects of Hydrogen Peroxide Injection into Left Testis on Malondialdehyde Level and Spermatogenesis in Left Testicular Tissue

**Figure 5:**
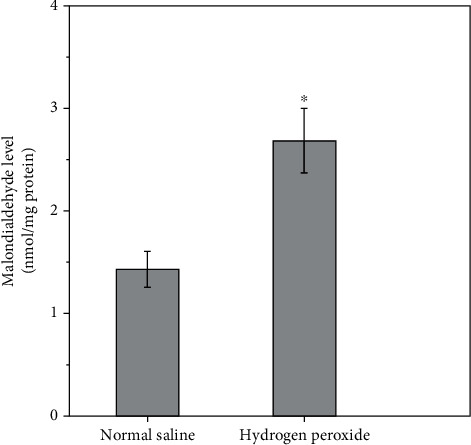
Left testicular malondialdehyde level in normal saline-injected and hydrogen peroxide-injected groups. All quantitative data are displayed as mean ± standard deviation (*n* = 10 each group). ^∗^*P* < 0.0001, compared with the normal saline-injected group.

**Figure 6:**
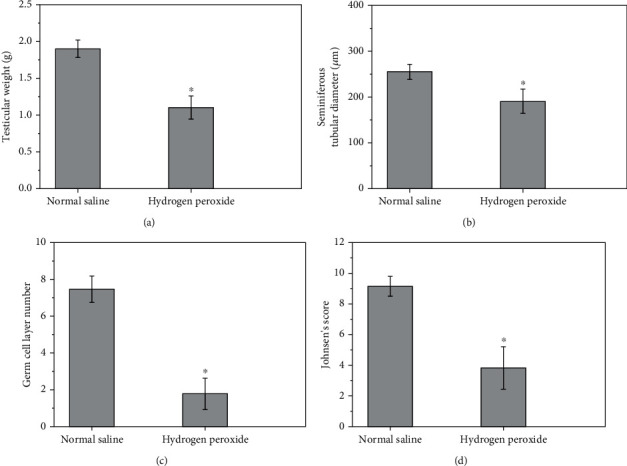
Left testicular weight (a), seminiferous tubular diameter (b), germ cell layer number (c), and Johnsen's score (d) in normal saline-injected and hydrogen peroxide-injected groups. All quantitative data are displayed as mean ± standard deviation (*n* = 10 each group). ^∗^*P* < 0.0001, compared with the normal saline-injected group.

**Figure 7:**
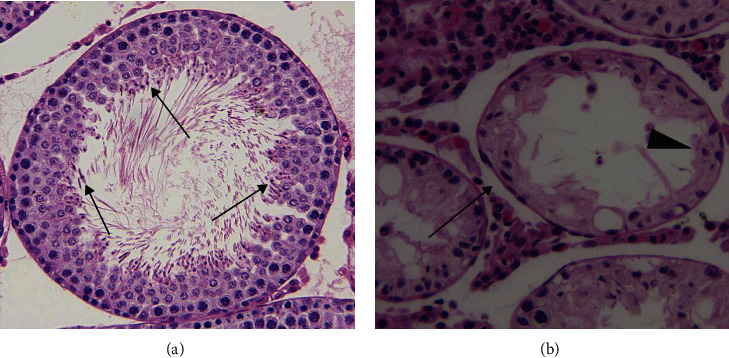
Light microscopy of hematoxylin and eosin stained sections of rat left testicular tissues in normal saline-injected and hydrogen peroxide-injected groups (original magnification, ×200). (a) The left testes of normal saline-injected group showed normal diameter of seminiferous tubule and germ cell layer number. There were many mature spermatozoa (↑) and an open tubular lumen in seminiferous tubule. (b) The diameter of seminiferous tubule (↑) and germ cell layer number (▲) were lower in left testes of the hydrogen peroxide-injected group versus normal saline-injected group (*P* < 0.0001). Mature spermatozoa were completely absent in seminiferous tubule.

Left testicular malondialdehyde level and spermatogenesis for normal saline-injected and hydrogen peroxide-injected groups are shown in Figures 5–7. A significant increase in left testicular malondialdehyde level was seen at four hours after injection of hydrogen peroxide into left testicular artery, as compared with the normal saline-injected group (*P* < 0.0001; Figure 5). The testicular weight, seminiferous tubular diameter, germ cell layer number, and Johnsen's score in left testes decreased significantly three months after injection of hydrogen peroxide, as compared with the normal saline-injected group (*P* < 0.0001; Figures 6 and 7).

## 4. Discussion

Testicular torsion is a common urological emergency that occurs in men younger than 25 years of age with an incidence of 1/4,000 [29]. An immediate surgical detorsion is required to secure survival of the torsional testis. If testicular torsion is not treated within six hours of symptom onset, it will result in testicular necrosis [2, 3]. Even if detorsion operation is performed within this time period, testicular atrophy may still occur [4–6]. Our study showed that testes were still viable after rats received two hours of left testicular torsion followed by detorsion. Nevertheless, severe damage of spermatogenesis was observed three months after detorsion. Testicular damage was characterized by significant reductions in testicular weight, seminiferous tubular diameter, germ cell layer number, and Johnsen's score (Figures 3 and 4). These findings are in keeping with the results of previous studies [30–33].

Testicular torsion/detorsion-induced damage is an ischemia-reperfusion injury. Ischemia-reperfusion induces the formation of large amounts of reactive oxygen species, such as hydrogen peroxide, superoxide anion, and hydroxyl radical [7–10]. Increased production of reactive oxygen species can cause peroxidation of cell membrane lipids and damage both DNA and protein function, leading to cellular dysfunction and death [11]. Reactive oxygen species, except hydrogen peroxide, are very difficult to measure directly due to their high reactivity and short life-span [34–36]. Malondialdehyde is a stable end product of lipid peroxidation in the cell membrane generated by reactive oxygen species and is widely used as a sensitive biomarker of reactive oxygen species [37, 38]. Our data indicated that the increased malondialdehyde level in the ipsilateral testes after unilateral testicular ischemia-reperfusion was accompanied by decreased spermatogenesis (Figures 2–4). These findings suggest that overgeneration of reactive oxygen species in the course of testicular ischemia-reperfusion is associated with testicular injury. To investigate whether reactive oxygen species can cause testicular damage, we injected hydrogen peroxide (a representative of reactive oxygen species) into left normal testis via the testicular artery. We found that hydrogen peroxide injection caused a significant increase in malondialdehyde level and a significant decrease in spermatogenesis in left testes, compared with the normal saline-injected group (Figures 5–7). These results confirm that reactive oxygen species are responsible for the disruption of spermatogenesis. Previous reports have indicated that the pharmacological agents, which can eliminate reactive oxygen species or reduce reactive oxygen species production, exert protective effect on ischemia-reperfusion injury in multiple organs, including heart, kidney, brain, and liver [39–42].

As a strong antioxidant, salvianolic acid B has the ability to attenuate ischemia-reperfusion injury in the heart, brain, liver, kidney, spinal cord, and skin flap [19–24]. This success led us to investigate the effect of salvianolic acid B on testicular ischemia-reperfusion injury in a rat model. We observed that a significant decrease of malondialdehyde level and a significant increase of spermatogenesis in ipsilateral testes occurred after salvianolic acid B treatment (Figures 2–4). These results indicate that salvianolic acid B has beneficial effect on testicular ischemia-reperfusion injury by decreasing reactive oxygen species content. It has been reported that salvianolic acid B can effectively protect against liver fibrosis in patients with chronic hepatitis B [43]. Salvianolic acid B showed no obvious side effects during the whole treatment [43]. Taken together, these findings suggest that salvianolic acid B has great potentialities to become a new and effective drug for the treatment of testicular ischemia-reperfusion injury. However, the exact mechanisms through which salvianolic acid B decreases reactive oxygen species content have not completely been clarified yet.

The xanthine oxidase is considered to be a primary source of reactive oxygen species formation in ischemia-reperfusion tissue [44–46]. Adenosine triphosphate (ATP) is used by cells as an energy source to keep cellular homeostasis. During the ischemic phase, cellular ATP production slows down because of decreased oxygen level in ischemic tissue [47]. The cellular energy failure leads to dysfunction of ATP-dependent pumps on the cellular surface, which causes influx of calcium ions into the cell [48]. The influx of calcium ions into the cell elevates intracellular calcium concentration [49, 50]. In cells, the accumulation of calcium ions activates a calcium-dependent protease that converts xanthine dehydrogenase to xanthine oxidase [49–51]. During ischemia, cellular ATP is catabolized through various intermediates to hypoxanthine, which accumulates in the cells [52–54]. A large amount of oxygen, together with blood, enters ischemic tissue during reperfusion. Xanthine oxidase uses hypoxanthine as a substrate and oxygen as a cofactor to generate both hydrogen peroxide and superoxide anion [55–57]. In the presence of iron, hydrogen peroxide reacts with superoxide anion to generate hydroxyl radical [55, 58, 59]. Therefore, an abundance of reactive oxygen species is produced during ischemia-reperfusion. Our study revealed that unilateral testicular ischemia-reperfusion significantly increased xanthine oxidase expression and malondialdehyde level and significantly reduced spermatogenesis in ipsilateral testes (Figures 1–4). These data suggest that upregulation of the xanthine oxidase expression in testicular tissue during testicular ischemia-reperfusion promotes reactive oxygen species outburst, leading to impairment in spermatogenesis. Our findings are in line with study results of Duman et al. [60]. Moreover, we found that salvianolic acid B treatment caused significant decreases in the xanthine oxidase expression and malondialdehyde level and caused a significant increase in spermatogenesis in ipsilateral testes (Figures 1–4). These findings support the idea that salvianolic acid B treatment downregulates the xanthine oxidase expression in testicular tissue and reduces reactive oxygen species generation, which results in an increase in spermatogenesis.

Several previous studies have demonstrated that salvianolic acid B at a dose of 10 mg/kg can protect rats from ischemia-reperfusion injury in the heart, brain, liver, and spinal cord [19–21, 23]. Hence, the therapeutic dose was selected in our rat model of testicular ischemia-reperfusion injury. In general, intravenous injection of drug takes effect more quickly than intraperitoneal injection. As a result, salvianolic acid B was injected intravenously in our and other studies [20, 21, 23]. Our study revealed that administration of salvianolic acid B (10 mg/kg) could significantly improve spermatogenesis in ipsilateral testes, compared with the testicular ischemia-reperfusion group, but the rescued spermatogenesis did not reach normal level (Figures 3 and 4). To obtain the optimal therapeutic effect of salvianolic acid B, its different doses and injection times should be determined with additional research.

Whether unilateral testicular ischemia-reperfusion has negative effects on the contralateral testis is a controversial issue. Some studies indicated that unilateral testicular ischemia-reperfusion could damage the contralateral testis [61–63], whereas others supported the opposite point of view [64, 65]. Our data showed that ipsilateral testicular xanthine oxidase protein expression, malondialdehyde level, and spermatogenesis were significantly changed after unilateral testicular ischemia-reperfusion, but these parameters in contralateral testes were not significantly affected (Figures 1–4). Therefore, we think that unilateral testicular ischemia-reperfusion does not cause impairment to the contralateral testis.

Previous study has shown that *Salvia miltiorrhiza* has protective effect on testicular ischemia-reperfusion injury [66]. The constituents of *Salvia miltiorrhiza* include more than 50 hydrophilic compounds which primarily have a phenolic acid structure (such as salvianolic acid B, salvianolic acid A, and protocatechuic aldehyde) and more than 30 lipophilic compounds which mainly have a diterpene chinone structure (such as isotanshinone I–II, cryptotanshinone, and tanshinone I–VI) [67]. In the present study, we investigated the effect of salvianolic acid B (a major component of *Salvia miltiorrhiza*) on testicular ischemia-reperfusion injury. Our study demonstrates that salvianolic acid B can reduce testicular ischemia-reperfusion injury (Figures 3 and 4). Whether the other components of *Salvia miltiorrhiza* have beneficial effect on testicular ischemia-reperfusion injury needs further research.

Many studies have displayed that salvianolic acid B can ameliorate ischemia-reperfusion injury in the heart, brain, liver, kidney, spinal cord, and skin flap [19–24]. In these studies, salvianolic acid B attenuated ischemia-reperfusion injury by antioxidative, anti-inflammatory and antiapoptotic mechanisms [19–24]. However, xanthine oxidase pathway was not involved in these mechanisms. Hence, in the current study, we investigated the effect of salvianolic acid B on testicular ischemia-reperfusion injury and related mechanism involving xanthine oxidase pathway. Our study showed that salvianolic acid B exerted protective effect on testicular ischemia-reperfusion injury by downregulating xanthine oxidase protein expression to inhibit reactive oxygen species formation (Figures 1–4).

A group receiving only salvianolic acid B without testicular ischemia-reperfusion can help researchers to evaluate a positive or negative effect of salvianolic acid B on testicular tissue and spermatogenesis. In our unilateral testicular ischemia-reperfusion + salvianolic acid B treatment group, we found that salvianolic acid B could attenuate ipsilateral testicular ischemia-reperfusion injury (Figures 3 and 4). However, salvianolic acid B had no effect on contralateral normal testis (Figures 3 and 4). Consequently, the group receiving only salvianolic acid B without testicular ischemia-reperfusion can be omitted in the current study.

Some investigators have demonstrated that sole usage of ketamine is safe and efficacious in anesthesia of rat testicular ischemia-reperfusion operation [68–70]. Therefore, ketamine was used solely in our surgical procedure. Our rat experiment also confirmed its efficacy.

Testicular torsion interrupts blood flow to the testis, which results in testicular ischemia. If salvianolic acid B is intravenously administered through the tail vein during testicular torsion, it will not enter testis to exert its protective effect. Surgical detorsion restores blood supply to the ischemic testis. Hence, we injected salvianolic acid B intravenously at reperfusion.

The proliferating cell nuclear antigen (PCNA) is involved in DNA replication and repair [71]. It is used as a cell proliferation marker [72]. The reduction in the PCNA expression in testicular germinal cells is an indicator for the reduction in spermatogenesis and proliferative activity [72]. The Ki-67 protein is also a cell proliferation marker. The two markers have been used in testicular ischemia-reperfusion injury studies [71–79]. In future study, we will investigate the effect of salvianolic acid B on the two markers in the rat testicular ischemia-reperfusion model.

## 5. Conclusion

In summary, we report for the first time that salvianolic acid B can attenuate testicular torsion/detorsion-induced ischemia-reperfusion injury. Salvianolic acid B improves testicular spermatogenesis by reducing reactive oxygen species generation by downregulating the xanthine oxidase protein expression. We hope that salvianolic acid B can become the first therapeutic agent for the treatment of testicular ischemia-reperfusion injury in clinical practice. However, further clinical trials should be performed to prove this point.

## Data Availability

All data used to support the findings of this study are included in the article.
